# Exercise improves pulmonary fibrosis and neurological symptoms via S100A12 inhibition

**DOI:** 10.3389/fimmu.2025.1583827

**Published:** 2025-06-27

**Authors:** Yan Sun, Meng Li, Jingmin Yuan, Wenrui Li, Hongli Quan, Yan Li, Zhenjing Kang, Hao Cheng, Hui Ren, Mingwei Chen

**Affiliations:** ^1^ Department of Respiratory and Critical Care Medicine, First Affiliated Hospital of Xi’an Jiaotong University, Xi’an, Shaanxi, China; ^2^ Department of Talent Highland, The First Affiliated Hospital of Xi’an Jiaotong University, Xi’an, Shaanxi, China; ^3^ Health Science Center, Yangtze University, Jingzhou, Hubei, China; ^4^ Department of Rehabilitation Medicine, First Affiliated Hospital of Xi’an Jiaotong University, Xi’an, Shaanxi, China

**Keywords:** pulmonary fibrosis, exercise, bioinformatics, lung function, neurological symptoms

## Abstract

**Background:**

Neurological symptoms are commonly observed in patients with idiopathic pulmonary fibrosis (IPF). However, the underlying mechanisms remain unclear. Although exercise has been shown to improve pulmonary fibrosis and quality of life in IPF patients, its effects on neurological symptoms in this population are not well understood. Furthermore, a robust animal model linking IPF with comorbid neurological symptoms has not yet been fully developed.

**Methods:**

Twenty-eight male C57BL/6J mice were divided into four groups: control, bleomycin (BLM), control + exercise, and BLM + exercise. Mice were administered BLM or saline (7.5 mg/kg), and the exercise groups underwent 45 min of treadmill training per day for 28 days. Behavioral tests (open-field test, sucrose preference test, tail suspension test, and forced swimming test) were performed on days 29–33. Histological analysis assessed pulmonary fibrosis, and biomarkers brain-derived neurotrophic factor (BDNF) and c-Fos were detected. Bioinformatics identified genes altered in IPF, exercise, and depression, validated by Western blotting.

**Results:**

BLM induced pulmonary fibrosis and aggravated neurological symptoms. Exercise significantly alleviated these symptoms and reversed the expression of BDNF and c-Fos. Bioinformatics analysis identified 28 genes upregulated in IPF and depression and downregulated by exercise. The S100A12 gene showed reduced expression in both lung and brain tissues in the BLM group and increased expression after exercise. Kyoto Encyclopedia of Genes and Genomes analysis revealed enrichment in the Interleukin 17 (IL-17) and Nucleotide-binding Oligomerization Domain (NOD)-like receptor signaling pathways.

**Conclusion:**

This study developed a mouse model and suggests that exercise may offer therapeutic benefits for both pulmonary and neurological symptoms in IPF. Shared molecular pathways may guide future therapies targeting both aspects.

## Introduction

Idiopathic pulmonary fibrosis (IPF) is a chronic, progressive and fatal disease, with a median survival of less than 4 years after diagnosis (1). Anxiety and depression, common mental disorders, frequently occur in patients with chronic, refractory diseases (2). Due to the high costs of treatment and the irreversible progression of the disease, IPF patients often experience anxiety, depression, and somatization. Studies indicate that 31% of IPF patients suffer from anxiety, whereas 23% experience depression (3). The presence of anxiety and depression in IPF patients not only affects their mental well-being but also has significant implications for their physical health and overall prognosis. Anxiety can intensify the perception of breathlessness, creating a vicious cycle where the physical symptom of dyspnea reinforces psychological distress, and vice versa (4–6). This heightened sense of breathlessness can lead to increased feelings of panic and helplessness, further deteriorating the patient’s quality of life. Depression, on the other hand, can impair the patient’s ability to adhere to treatment regimens, engage in necessary physical rehabilitation, and maintain social connections (7, 8). The interplay between the physical burden of IPF and the psychological impact of anxiety and depression creates a complex challenge for both patients and healthcare providers, underscoring the importance of addressing mental health in the comprehensive care of IPF patients.

Recent studies have identified shared genetic variants between IPF risk loci on chromosomes 8/17 and brain imaging phenotypes, linking the protein DEPTOR to cortical thinning and potential associations with depression/cognitive decline (9). In addition, research has shown that the expression of ionized calcium-binding adaptor molecule (Iba1) was activated in the hippocampus of bleomycin (BLM) instillation mice, indicating the involvement of microglial activation and inflammatory factors in the development of depressive and anxiety-like behaviors in the context of BLM-induced pulmonary fibrosis. This effect is further exacerbated by intermittent hypoxia, which may contribute to the pathophysiology of these psychological symptoms (10).

Although clinical treatment strategies of pulmonary fibrosis have advanced rapidly, drug therapies remain the primary approach (11). In addition to established medications like Pirfenidone and Nintedanib, novel drugs targeting fibroblasts, macrophages, and stem cell therapy are currently undergoing clinical trials (12). Interestingly, fluvoxamine, a commonly used medication for treating anxiety and depression, exhibits anti-fibrotic effects by inhibiting the cyclic GMP-AMP synthase-stimulator of interferon genes (cGAS-STING) pathway and related signaling pathways (13), making it a potential effective treatment for IPF. Notably, patients with IPF are increasingly benefiting from supportive therapies such as pulmonary rehabilitation (PR), management of comorbidities, and education (14). Exercise training, a cornerstone of PR, has been shown to directly alleviate dyspnea and fatigue in patients with chronic lung diseases (15). Recent randomized controlled trials have demonstrated that exercise improves lung function and quality of life in patients with IPF (16). However, the essential role of exercise in addressing both IPF and the associated anxiety and depression remains poorly understood.

Currently, the BLM-induced mouse model is the classical model widely used to study the physiological mechanisms of pulmonary fibrosis. This model successfully mimics the phenotypes of pulmonary fibrosis, airway inflammation, and impaired lung function (17, 18). However, most existing animal models of pulmonary fibrosis primarily focus on the physical aspects of the disease, with limited exploration of the concurrent mental health comorbidities, such as anxiety and depression, often seen in patients with IPF. Notably, a study conducted in 2023 (10) demonstrated that exposure to hypoxic environments significantly exacerbates anxiety and depressive behaviors in BLM-induced IPF mice. This finding highlights the potential interaction between respiratory dysfunction and mental health in IPF, suggesting that the physiological stress associated with hypoxia may exacerbate the psychological distress experienced by patients with IPF.

Building on this, the present study aimed to address these gaps by exploring the phenotypic manifestations in mice induced with varying doses of BLM during the modeling process. This approach established a model that not only replicates the typical features of IPF but also exhibits anxiety- and depression-like behaviors. After 28 days of exercise intervention, the researchers assessed pulmonary fibrosis severity, anxiety-depression-like behaviors, biomarkers, and lung function in both control and BLM-treated groups. Furthermore, bioinformatics analysis was employed to identify key target genes affected by exercise, which may influence both pulmonary fibrosis and the associated anxiety and depression, providing valuable insights for the diagnosis, treatment, and evaluation of IPF and related mental health disorders.

## Materials and methods

### Animal model

Mice were anesthetized with 1% pentobarbital sodium (80 μL/10 g) and then were administered a single intratracheal injection of BLM hydrochloride (Selleck, #S1214) dissolved in normal saline (NS) was administered. Control mice were given 0.9% NS following the same procedure. The maximum volume administered via tracheal intubation in mice should not exceed 50 μL to prevent suffocation (10, 19–21).

For the establishing of the BLM-induced fibrosis model, the researchers initially referred to the manufacturer’s recommended dose of 5 mg/kg, as outlined in the product datasheet (Selleck, #S1214). However, a review of the existing literature revealed that various studies employed different doses of BLM, ranging from 1 to 4 units/kg (18). This variability was likely influenced by factors such as the brand of BLM reagent, its concentration, and differences in the animal batches used, all of which could contribute to the observed dose variability.

To optimize the dosing regimen, preliminary experiments were performed to evaluate the efficacy and safety of different doses in their specific experimental conditions. On the basis of these efforts, a dose gradient of BLM at 2.5 mg/kg, 5 mg/kg, and 7.5 mg/kg was selected. This range allowed for the evaluation of dose-dependent effects on fibrosis progression while ensuring the animal welfare and the reproducibility of the results.

To explore the effects of varying BLM doses on the development of an IPF mouse model with comorbid anxiety and depression-like behaviors, this study tested three different drug concentrations. Hematoxylin-eosin (HE) staining and the Ashcroft score were used to quantify the extent of fibrosis. Survival curves were analyzed to evaluate the stability of the animal models, and the open-field test (OFT) was conducted to assess anxiety and depression-like behaviors in the mice.

### Modeling administration and exercise training

A total of 28 C57BL/6J male mice (8 weeks, 23.4 0.7 g) purchased from Charles River Company (Beijing, China) were randomly separated into four groups (control, control + exercise, BLM, and BLM + exercise) for the next experiment after acclimating to the environment for a week under specific pathogen–free conditions (temperature of 20°C–25°C, relative humidity of 45%–60%, and 12– h light–12-h dark cycle).

Mice were given BLM (7.5 mg/kg; Selleck, #S1214) or NS by endotracheal instillation after anesthesia on day 0. After recovered for 24 h, mice in the control + exercise group and the BLM + exercise group were started to do treadmill exercise 45 min, at a 0° slope and a speed of 12m/min for 28 consecutive days [TECHMAN small animal treadmill (FT-201)]. Body weight was measured every other day, and anxiety-depression-like behaviors were performed on days 28–33.

All mice were sacrificed on day 34, and the lung and brain tissues were collected for subsequent experiments. Under anesthesia, the mouse thoracic cavity was opened to expose the heart. A 10-mL syringe was inserted into the left ventricle, and the right atrial appendage was incised to allow blood drainage. Approximately 30 mL of NS was perfused, and limb and tail twitching/stiffening was observed during the procedure. Successful perfusion was confirmed by the pale, distended appearance of the lungs and liver. Lung tissue was then carefully dissected on ice, and the lobes were separated and labeled as left upper, left lower, right upper, right middle, and right lower. The right middle lobe was fixed in 4% paraformaldehyde for H&E, Masson’s trichrome, and immunohistochemical staining, whereas the remaining lobes were stored at −80°C for subsequent analysis (22).

### Behavioral tests

#### Open-field test

OFT were utilized to assess exploration, movement, and anxiety (23). The square cage (length of 50 cm, width of 50 cm, and height of 35 cm) was used, and, then, each mouse is gently placed in the center square area of the cage (center area). Computer-automated animal activity video-tracking system (VisuTrack 3.0.0.1) was conducted to record the activities of mice for 5 min to quantify the behaviors. Velocity, time spent, and the frequency of entries in the center arena of the cage were measured in the OFT.

#### Sucrose preference test

Sucrose preference test (SPT) was used to assess the anhedonia of rodents as in previous studies (24–26). Mice were first trained to consume 1% sucrose solution before the formal experiment and then exposed to a bottle of 1% sucrose solution and water for 60 min after depriving of water and food for 24 h, and the bottle positions were changed every 30 min. The sucrose preference (%) = sucrose intake/(sucrose intake + water intake) × 100%.

#### Tail suspension test

The tail suspension test (TST) was used to measure depressive/despair-like behavior in rodents by measuring the time of immobility during tail suspension (27) as described previously (24, 26). Immobility is defined as a behavior without struggling or lacking movement of any limb or body other than respiratory movement. Mice were individually suspended 6 min by the tail from a vertical bar on the top of a box (30 cm × 30 cm × 30 cm). The immobility time was recorded during the last 4 min.

#### Forced swimming test

The depression-like behavior in rodents was evaluated by forced swimming test (FST) (26, 28). Each mouse was forced to swim independently for 6 min in a plastic cylinder (height of 50 cm and diameter of 20 cm) containing fresh water up to a height of 23 cm at 25°C. Duration of the immobility in the last 4 min and the latency to immobility (motionless for at least 1 s) of mice were recorded and analyzed using a computer-automated animal activity video-tracking system (VisuTrack 3.0.0.1).

### Measurement of fibrosis-related markers

Lung tissues were fixed in 4% paraformaldehyde, embedded in wax, and sectioned at 5 µm. HE and Masson’s trichrome were conducted according to the standard procedures. The microscope (Leica, Germany) was used for morphological observation. Ashcroft score was conducted to evaluate the BLM-induced lung fibrosis based on stained histological samples by visual assessment (29). Collagen contents in lung sample were measured by using hydroxyproline (HYP) assay (RXJW202684M, Ruixin Bioengineering Institute, China) according to the manufacturer’s instructions.

### Immunofluorescence analyses

The primary antibodies (**Supplementary Table S1**) were added onto the processed paraffin sections and then incubated at 4°C for 12 h and, subsequently, incubated with the secondary antibody for 120 min at room temperature. 4’,6-Diamidino-2-phenylindole (DAPI) were used as nuclear stain. Images were collected under an inverted fluorescence microscope.

### Pulmonary function testing

Pulmonary function, including breaths per minute (BPM), tidal volume, peak expiratory flow (PEF), and peak inspiratory flow (PIF) of mice in each group, was detected on the DSI Buxco platform by using respiratory and pulmonary function test system (UPWARDS TEKSYSTEMS LTD.) according to the instrument operating instructions.

### Datasets collection

Microarray and RNA-seq datasets based on the whole blood from the Gene Expression Omnibus (GEO) database (https://www.ncbi.nlm.nih.gov/geo/) were collected to explore the shared genetic interrelations among male patients with IPF (GSE28042, 52 IPF vs. 12 control), exercise (GSE111552, 16 exercise vs. 16 control), and major depressive disorder (GSE98793, 32 MDD vs. 16 control) (30).

### Differential expression analysis

The differentially expressed genes (DEGs) were respectively identified by “limma” package with logFC > 0.1, *P* < 0.05, in the three datasets, and the results were visualized as volcano map. In this study, DEGs that are upregulated in IPF and MDD and DEGs that are downregulated post exercise were identified as disease-causing genes that promote disease progression. Venn diagram was carried out to identify the overlapping genes affected by IPF, MDD, and exercise, and the heatmap was utilized to present the expression of overlapping genes in every dataset.

### PPI network construction and correlation analysis

The online platform STRING database (https://www.string-db.org) were conducted for protein-protein interaction (PPI) network and correlation heatmap based on overlapping genes (31), with cutoff values of more than 0.15.

### Kyoto Encyclopedia of Genes and Genomes pathway enrichment analysis

Kyoto Encyclopedia of Genes and Genomes (KEGG) pathway enrichment analysis was performed using the online tool SangerBox 3.0 (http://vip.sangerbox.com/home.html) to identify the roles of DEGs in various biological processes and the biological pathway that they are involved in. The results of the analysis are presented in the form of circle plots and bubble plots, with a *P*-value of <0.05 indicating statistically significant differences.

### Statistical analyses

ImageJ software was used to count the number of positive cells for c-Fos and to analyze the immunofluorescence intensity of BNDF. R (4.0.1) and GraphPad Prism 9.0 were performed to data analysis. The Kolmogorov–Smirnov normality test and the variance homogeneity test were performed. Data were presented as mean ± standard deviation (SD). Statistical analysis was conducted using ANOVA that was carried out for data analysis. *P* < 0.05 was considered statistically significant.

## Results

### Mouse model of IPF with comorbid anxiety and depression

HE staining of lung tissue from mice with BLM-induced fibrosis at different doses showed that the fibrosis effect was most pronounced in the dosage group of 7.5 mg/kg (**Supplementary Figures S1A, B**). Additionally, there was no significant different in mortality rated among the three groups (**Supplementary Figures S1C**). Furthermore, a preliminary OFT revealed that mice in the dosage group of 7.5mg/kg exhibited anxiety and depression-like phenotypes (**Supplementary Figures S1D–J**).

### Exercise ameliorates BLM induced inflammation and fibrosis

**Figure 1:**
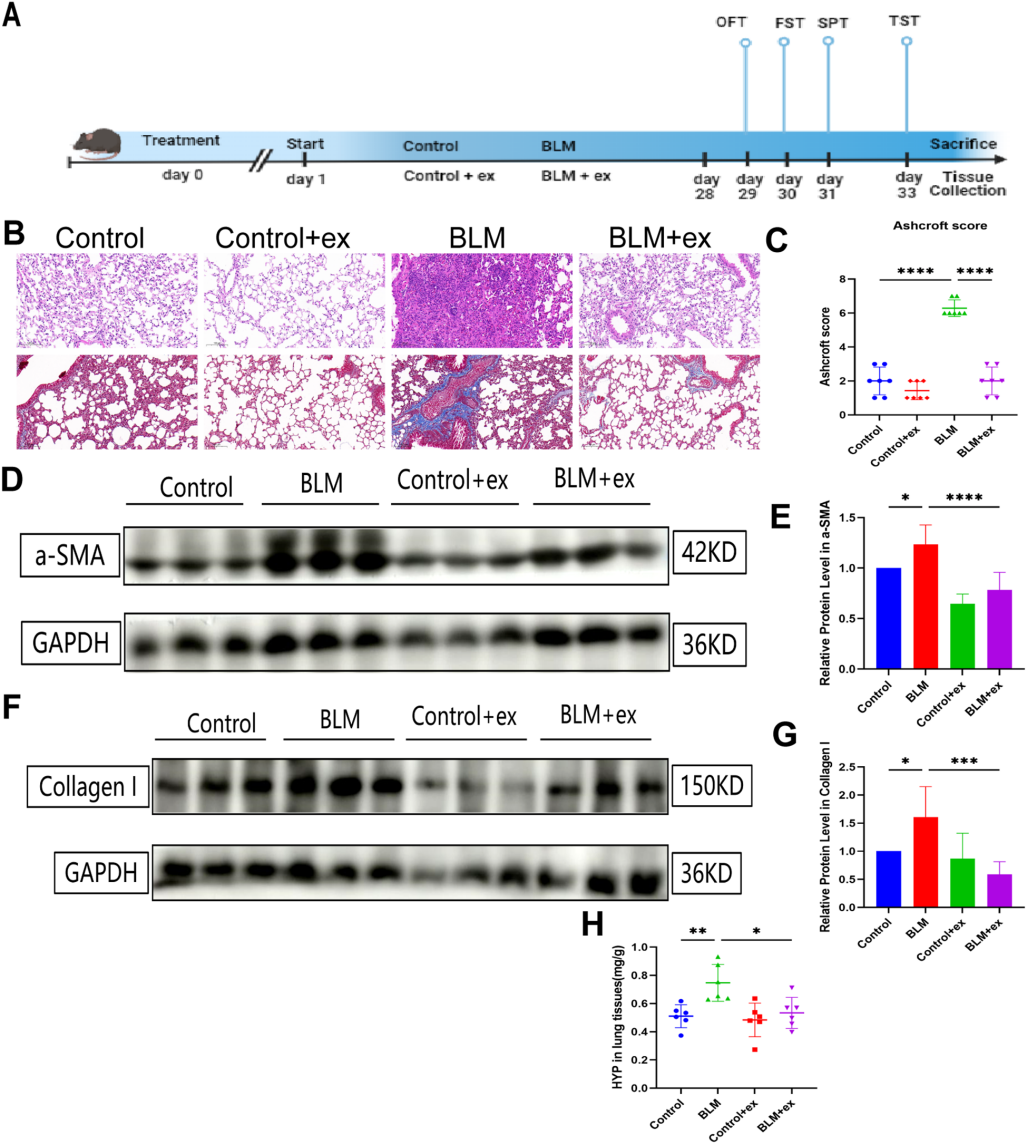
Exercise alleviates BLM‐induced lung fibrosis. **(A)** Experimental flowchart of the present study. Representative images of hematoxylin-eosin and Masson’s trichrome staining **(B)** and comparison of Ashcroft score **(C)** of mice in different groups. Expression of α-SMA **(D, E)** and collagen I **(F, G)** in different groups.**(H)** The 839 expression of HYP in lung tissues. Scale bars, 200 μm; * *P*<0.05; **P<0.01; ***P<840 0.001; ****P < 0.0001.

The experimental flowchart was shown in **Figure 1A**. Histopathological evaluation showed that the degree of alveolar structure disorder, alveolar edema, pulmonary septum thickening, and inflammatory cell infiltration were more severe in the BLM-induced model group than those in the control group (**Figure 1B**). Ashcroft score was also increased in the group treated with BLM (**Figure 1C**). To investigate the effect of exercise on the BLM-induced pulmonary fibrosis, the study exposed BLM-tracheal injected mice to exercise 28 days; as shown in **Figures 1B, C**, the staining results displayed that exercise could significantly alleviate the fibrosis caused by BLM and was accompanied by downregulation of Ashcroft score. Exercise also significantly reduced the expression of fibrosis markers α-SMA and collagen I (**Figures 1D–G**). Additionally, lung tissue HYP content analysis revealed that, compared to the control group, the BLM group exhibited a significant increase in the HYP levels. However, following exercise, the HYP content was significantly reduced, further supporting the protective effects of exercise against BLM-induced fibrosis (**Figure 1H**).

### Exercise reduces the expression of biomarkers of anxiety and depression

**Figure 2:**
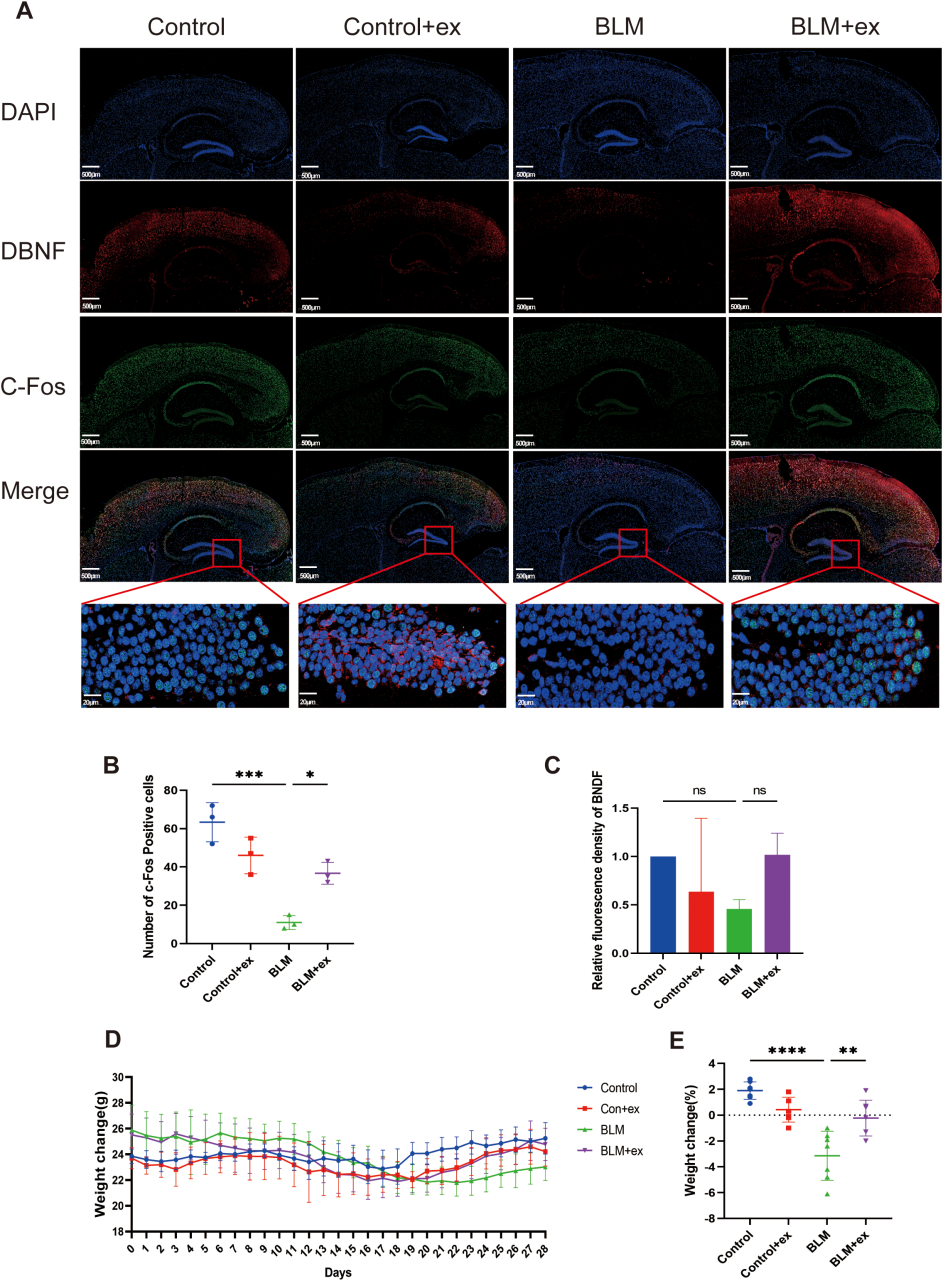
Exercise reduces the expression of biomarkers of anxiety and depression and improved the weight loss of the BLM-induced group. **(A)** Representative immunofluorescence images of DBNF (red) and c-Fos (green) of in mice brain tissue. **(B, C)** Statistical graphs of DBNF and c-Fos. Scale bars, 500 μm. Body weight change curve **(D)** and statistical graphs **(E)** in mice. * *P*<0.05; **P<0.01; ***P<0.001; ****P < 847 0.0001, ns P>0.05.

Brain-derived neurotrophic factor (BDNF) and c-Fos were considered classic biomarkers associated with anxiety and depression; herein, the distribution and expression of the above two biomarkers in brain after exercise training were measured by immunofluorescence staining to evaluate the effect of BLM on anxiety and depression in mice and the improvement of exercise. As shown in **Figures 2A–C**, compared to those in the control group, BDNF and c-Fos were found to be downregulated in the dentate gyrus (DG) of the hippocampus in the BLM-induced fibrosis mice. However, after exercise, there was an increase in BDNF expression, although the difference was not statistically significant. On the other hand, c-Fos expression was significantly upregulated in the exercise group, particularly in the DG region of the hippocampus.

### Exercise ameliorated BLM-induced weight loss in mice

**Figure 2D** presented the effects of BLM treatment and exercise exposed on mice body weight change during the experimental period. The weight of mice treated with BLM began to decrease from the seventh day and reached the lowest value on the 21st day. Although the weight of mice exposed to exercise downregulated in the early stage, it increased significantly from the 21st day, whereas the weight of mice in the control group was relatively stable and did not show significant fluctuations. As shown in **Figure 2E**, the mice in the BLM group lost significantly more weight than those in the control group, whereas the weight loss of mice in the BLM + exercise group was lower than that in the control + exercise group.

### Exercise improved depressive and anxiety-like behaviors in the BLM-induced pulmonary fibrosis mice

**Figure 3:**
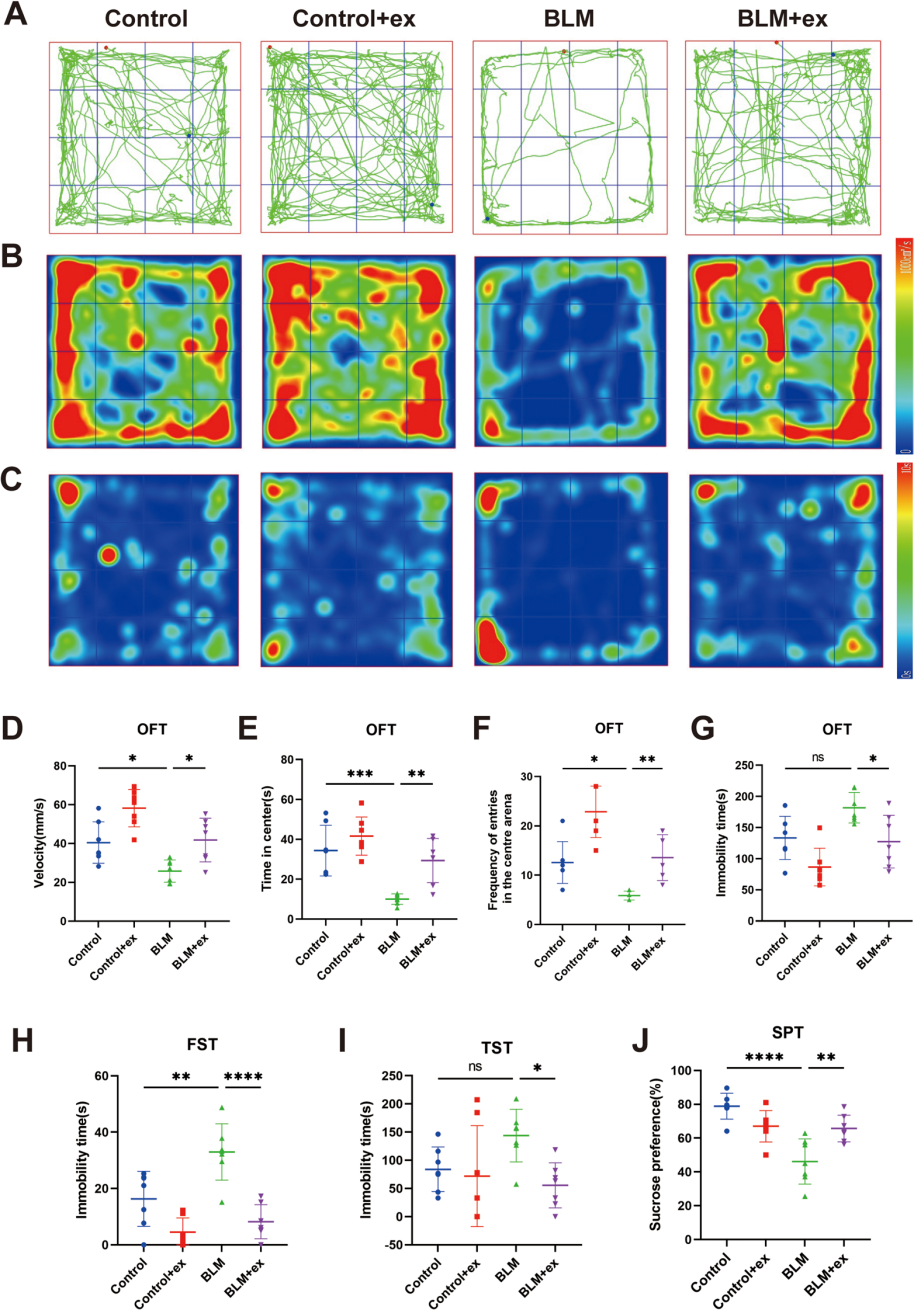
Exercise improved depressive and anxiety-like behaviors in the BLM-induced pulmonary fibrosis mice. Motion trajectories **(A)**, motion heatmap **(B)**, and residence time heatmap **(C)** in the OFT of mice. Analysis of the movement velocity **(D)**, the time spent of entries into the center **(E)**, the frequency of entrance in the central area **(F)**, and the immobility time **(G)** in the OFT of mice. Analysis of FST **(H)**, TST **(I)**, and SPT **(J)** of mice in different groups. BLM, bleomycin; OFT, open-field test; TST, tail suspension test; FST, forced swimming test; SPT, sucrose preference test. ^*^ *P* < 0.05, ^**^ *P* < 0.01, ^***^ *P* < 0.001, ^****^ *P* < 0.0001, and ns *P* > 0.05.

The roadmap (**Figure 3A**) and heatmap (**Figure 3B**) of movement and the heatmap of immobility time (**Figure 3C**) were measured in OFT. The results showed that the movement velocity (**Figure 3D**), time in center (**Figure 3E**), and frequency of entries in the central area (**Figure 3F**) were significantly decreased in the BLM model group, whereas the above behaviors of mice in the exercise intervention group were significant improved (**Figures 3D–F**). Exercise also significantly reduced the increase of immobility time caused by BLM in mice (**Figure 3G**). In addition, immobility time in FST (**Figure 3H**) and TST (**Figure 3I**) increased in the BLM-treated group compared to that in control group, which was significantly reduced after exercise. The mice in the BLM group also showed a significant preference for sucrose after exercise (**Figure 3J**).

### Exercise improved lung function of mice in the BLM group

**Figure 4:**
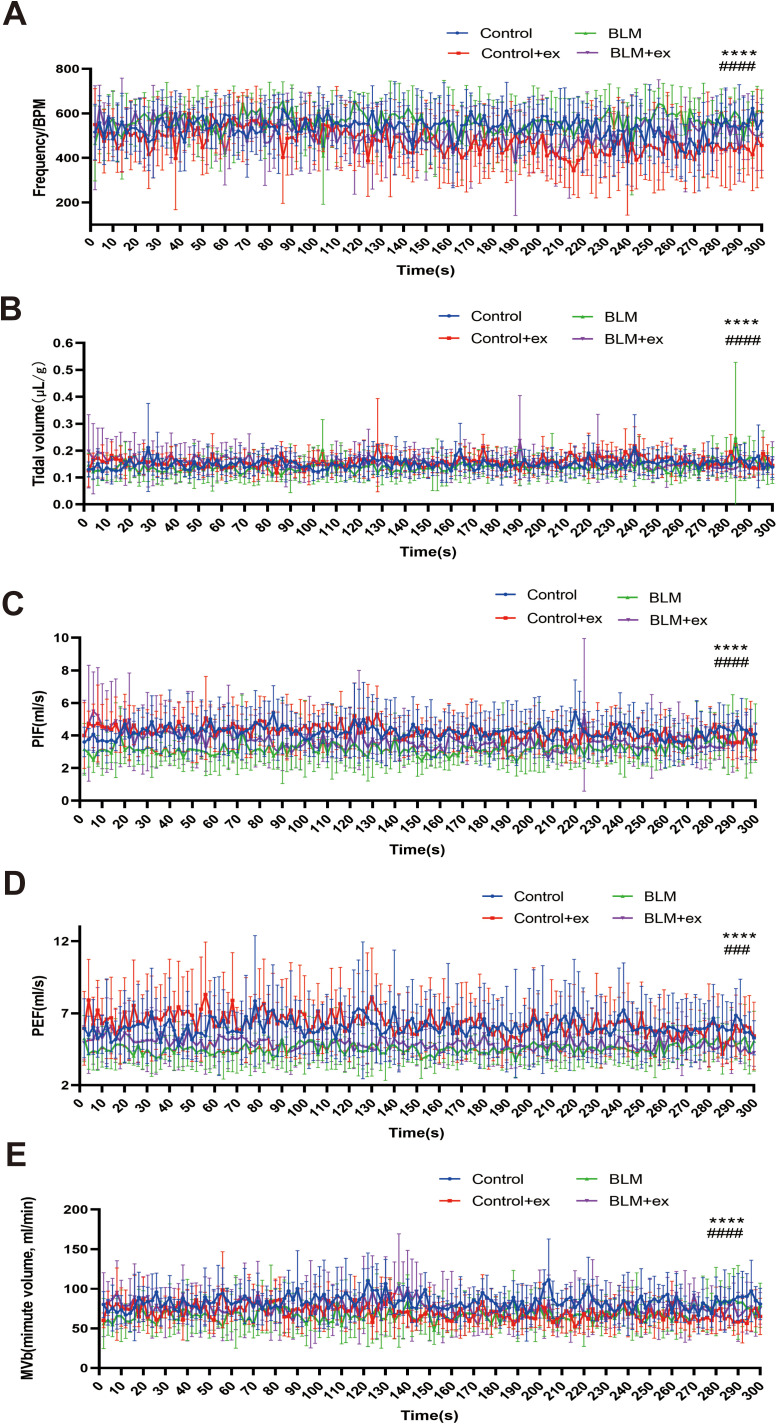
Exercise improved lung function of mice in the BLM group. Analysis of tidal BPM **(A)**, tidal volume **(B)**, PIF **(C)**, PEF **(D)**, and MVb **(E)** among four groups. BLM, bleomycin; BPM, breaths per minute; PEF, peak expiratory flow; PIF, peak inspiratory flow; MVb, minute volume. ^****^ *P* < 0.0001, ###*P* < 0.001, and #### *P* < 0.0001.

Compared to mice in the control group, the respiratory function was significantly impaired in the BLM-induced pulmonary fibrosis model of mice, which was mainly manifested as increased of respiratory rate (**Figure 4A**) and decreased of tidal volume (**Figure 4B**). Moreover, the PIF (**Figure 4C**), PEF (**Figure 4D**), and minute volume (**Figure 4E**) also showed a downward trend in mice treated with BLM. In contrast, the group of mice that received the exercise intervention showed significant improvements in respiratory function, as evidenced by decreased respiratory rate, increased tidal volume, PIF, PEF, and minute volume (**Figures 4A–E**).

### Identification of core genes affecting IPF, depression, and exercise

**Figure 5:**
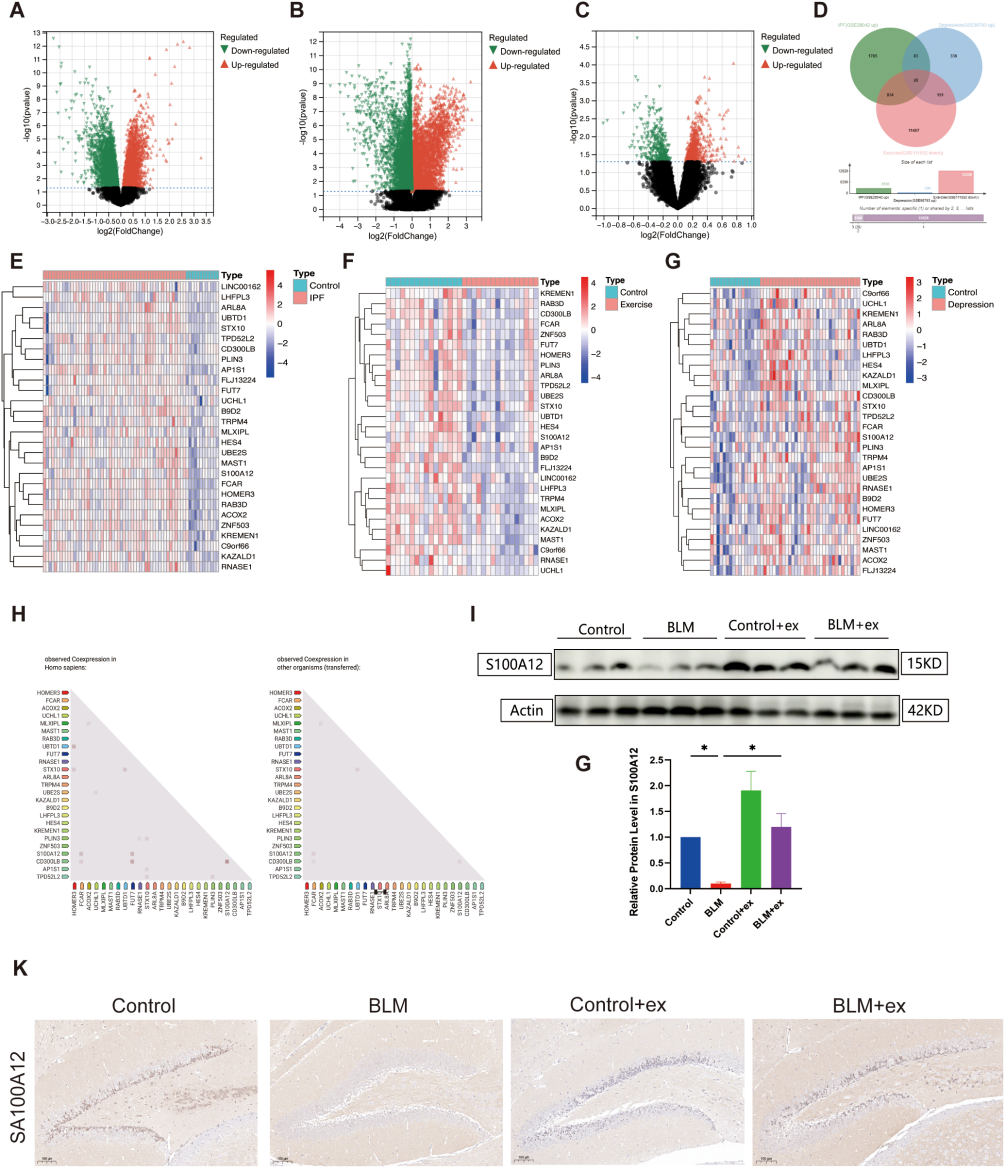
Bioinformatics analysis to identify intersection genes. Volcano map of DEGs in GSE28042 (IPF, **A**), GSE111552 (exercise, **B**), and GSE98793 (depression, **C**) datasets. Venn diagram identified intersection DEGs in the three databases **(D)**. Heatmap of 28 intersection genes in GSE28042 **(E)**, GSE111552 **(F)**, and GSE98793 **(G)** datasets. Differentially expressed genes (DEGs). Correlation analysis among 28 intersection genes based on STRING database **(H)**. Expression of S100A12 in in mice lung tissue **(I, J)**. Representative IHC images of S100A12 in mice brain tissue **(K)**. Scale bars, 100 μm. ^*^ *P* < 0.05.

As shown in **Figure 5**, volcano graph showed that there are 5,914 DEGs in IPF (2,830 upregulated and 3,084 downregulated; **Figure 5A**), 16,713 DEGs in exercise (4,193 upregulated and 12520 downregulated; **Figure 5B**), and 1,014 DEGs in depression (598 upregulated and 416 downregulated; **Figure 5C**). To explore the pathogenic genes that promote pulmonary fibrosis and depression, a Venn diagram was conducted and identified 28 intersection genes that increased in IPF, depression, and decreased in exercise (**Figure 5D**), and the results were visualized as heatmap (**Figures 5E–G**). Correlation analysis found significant correlation among some members of these 28 genes (**Figure 5H**). **Figures 5I, J** showed that S100A12 expression was significantly reduced in BLM-induced fibrotic tissue, which was increased after exercise. Similarly, S100A12 was decreased in the hippocampus of BLM-treated mice, whereas exercise upregulated its expression in the hippocampus (**Figure 5J**).

### Identification of signaling pathway

**Figure 6:**
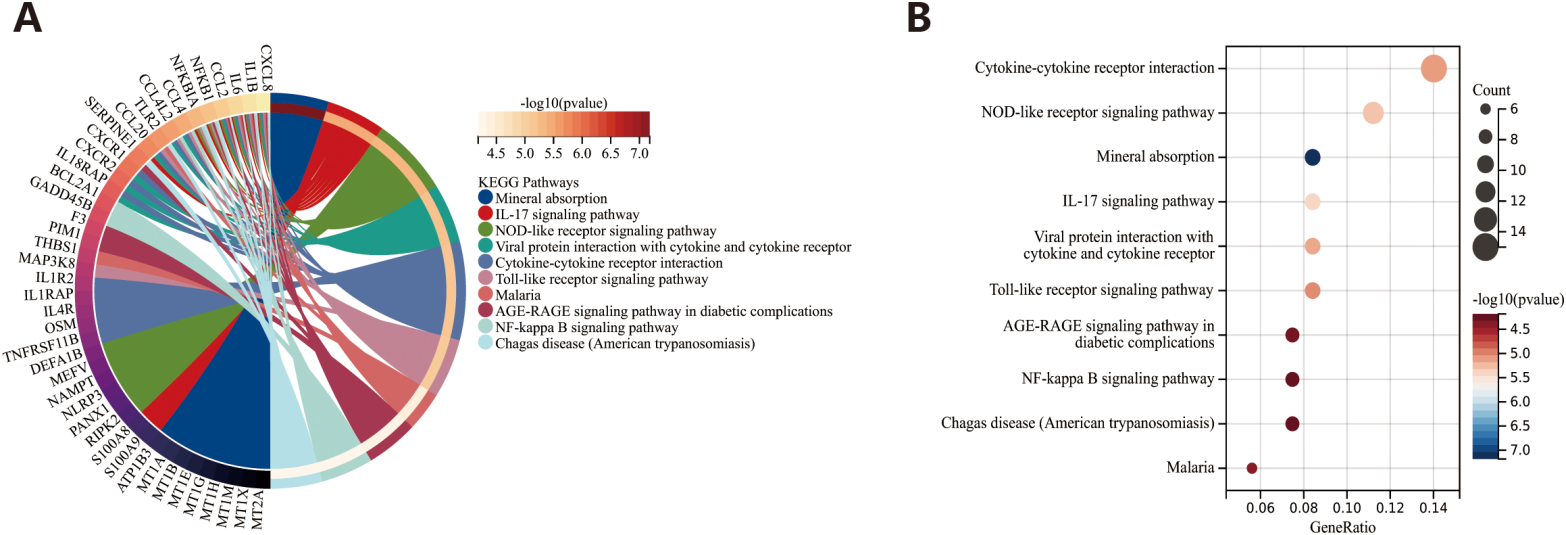
KEGG analysis to identify signaling pathway. **(A)** Pathway diagram. **(B)** Bubble chart.

In this study, the researchers performed a correlation analysis on the gene expression data from the dataset GSE32537 and identified genes significantly correlated with S100A12. By setting the absolute value of the correlation coefficient (R) greater than 0.5 as the screening criterion, 231 genes highly correlated with S100A12 were selected. Subsequently, KEGG enrichment analysis on the selected gene set was conducted. The KEGG enrichment analysis revealed that these genes were primarily enriched in pathways such as mineral absorption, IL-17 signaling pathway, NOD-like receptor (NLR) signaling pathway, viral protein interaction with cytokine and cytokine receptor, and cytokine–cytokine receptor interaction (**Figures 6A, B**).

## Discussion

Interstitial lung diseases (ILDs) comprise a variety of diseases characterized by progressive interstitial inflammation and/or fibrosis, leading to restricted physiological limitations and impaired gas exchange, reduce quality of life, and cause dyspnea in patients (32). IPF is one of the most common ILDs, with a very high mortality rate, and research shows that the median survival time is less than 3.9 years (33). Although anti-fibrotic drugs have been shown to slow disease progression and improve survival, there is currently no cure (11). Lung transplantation is another option for advanced IPF, improving survival and quality of life (34).

Mental health–related diseases are the leading cause of increased disability and impaired quality of life among older people and patients with chronic diseases worldwide. Observational studies estimate high prevalence of moderate to severe depression (more than 50%) and anxiety (40%) among patients diagnosed with IPF (35). Dyspnea, cough, sleep disturbance, loss of independence, social isolation, and fear of imminent death due to progressive and unpredictable course of the disease are the main causes of ultimately depression. Conversely, depression and anxiety can also contribute to adverse clinical outcomes and disease severity in patients with IPF (36). This raises the possibility that treating depression may improve the quality of life and reduce the burden of symptoms in IPF patients, or vice versa.

PR, including active exercise, passive exercise, and muscle building, is an effective intervention that can significantly improve exercise tolerance, symptoms, and quality of life in patients with IPF (37, 38). Previous studies have also demonstrated that exercise reduces suicide attempts in those with mental or physical illness, emphasizing the utilization of exercise to improve mental health outcomes (39). Existing literature supports the idea that exercise positively impacts both physical and mental health, particularly in individuals with chronic conditions such as Parkinson’s disease (40), spinal cord injury (41), and schizophrenia spectrum disorders (42). Additionally, exercise interventions have been linked to improvement in aerobic capacity, depression, and health-related quality of life, following hospital discharge in survivors of critical illness (43). However, it remains unclear whether exercise can improve pulmonary fibrosis and related anxiety and depression symptoms.

This study investigated the effects exercise intervention on pulmonary fibrosis in mice and demonstrates a significantly reduction in both histological damage and anxiety/depressive behaviors. The therapeutic effects of exercise are considered through two primary mechanisms: direct effects on the central nervous system and indirect improvements through lung function enhancement. A study has shown that exercise promotes neurogenesis in critical areas of the hippocampus, including the DG and cornu ammonis, which contributes to improved mental health and plays a significant role in preventing age-related brain volume decline (44).

BLM induced the inflammatory cell infiltration, collagen increased in interstitial of mice, and exercise training remarkably reduced the collagen aggregation and inflammation. Consistent with these findings, Prata et al. (45) found that exercise reduced lung injury in BLM-treated mice, manifested by reductions in connective tissue, type I collagen, type II pneumocytes, and pulmonary surfactant-associated protein A (SP-A). These results confirm that exercise improves the progression of pulmonary fibrosis at the histopathological level, and previous studies illustrated that it may be partly related to the promotion of attenuation of 5-hydroxytryptamine/protein kinase B (5-HT/Akt) (46) and the suppression of Transforming growth factor beta 1 (TGF-β1) / Smad proteins and Low-density lipoprotein receptor-related protein 6 (LRP-6)/β-catenin signaling pathways (47).

According to the neurotrophic hypothesis, BDNF may contribute to the development of depression, because its low expression was associated with atrophy of the brain areas involved in emotion (48). BDNF may affect the course of depression via brain-gut-microbiota axis, neural, Cyclic adenosine monophosphate (cAMP) response element-binding protein (CREB)/BDNF, and immune pathway (49). The immediate-early gene c-Fos, associated with the synaptic plasticity mechanism of depression, is activated quickly and briefly in response to a variety of cell stimuli and, in turn, controls the expression of some late response genes, such as BDNF and further regulates various cell reactions (50). On the other hand, ΔFosB exhibits sustained expression in response to chronic stimuli, specifically in the ventral hippocampus, and plays a fundamental role in mediating long-term changes associated with chronic stress. ΔFosB’s role extends to regulating the activity of glutamatergic neurons within this region, influencing behaviors related to both depression- and anxiety-like responses to stress (51). Thus, the integration of the roles of c-Fos and ΔFosB provides a more comprehensive understanding of the rapid and sustained responses of the nervous system to stress, opening new possibilities for targeted therapies aimed at correcting the long-term changes that underlie mood disorders like depression.

To clarify the morphological and functional changes of depression-related major brain regions, the expression of BDNF and c-Fos in the four groups was investigated, and the results showed that BNDF and c-Fos were remarkably reduced in the brain, especially in DG of the hippocampus. Exercise intervention reversed the expression of both genes. Similar to our findings, previous studies have shown that depression-like behavior is associated with impaired neuroplasticity, particularly neuronal atrophy, and synaptic loss in the medial prefrontal cortex (mPFC) and hippocampus (52). Notably, our study confirmed that there were significant expression differences of BDNF and c-Fos specifically in the DG region of the hippocampus, which aligns with previous research conclusions (53, 54). However, although we observed a downward trend in BDNF expression in the DG, the difference did not reach statistical significance. Future experiments with larger sample sizes could further validate this trend. These results suggest that the hippocampus, particularly the DG region, is key brain area through which BLM and exercise exert their effects on depression.

Notably, significant changes in depression and anxiety behaviors were also found between the different groups in the present study. Specifically, compared with control mice, the decrease in the time spent and the frequency of entries in center arena in OFT and the percent of sucrose preference in SPT and the increase in immobility time of the TST were observed in the BLM group. All the above symptoms raised by BLM were improved after exercise. Moreover, mice with anxiety and depression showed a decrease in body weight and improvement in lung function. This was consistent with the results of some clinical studies (10, 16, 55, 56).

Subsequent bioinformatics analysis identified 28 core genes that are upregulated in pulmonary fibrosis, depression, and downregulated after exercise, and the PPI network showed correlations between some of these genes. Of all the intersecting genes, ZNF503 (57), S100A12 (57), RNASE1 (58), LHFPL3 (59), TRPM4 (60), and UCHL1 (61) have been confirmed to be associated with depression, and, among these six genes, only UCHL1 (62) and S100A12 (63) are involved in the progression of pulmonary fibrosis. Epigenetic silencing of UCHL1 prevented the upregulation of COL1A1 induced by TGF-β1 in lung epithelial cells (62). Elevated UCHL-1 concentrations are associated with the deterioration of neurobehavioral symptoms (61). Previous studies have not verified the expression of UCHL1 in the fibrosis model induced by BLM (62), which may be due to the different potential molecular mechanisms of different carcinogenic fibrotic drugs.

Consistent with this study, S100A12 is reported to be most clinically diagnosable biomarker upregulated in depression based on machine learning algorithms (57), whereas, which was found upregulated in blood and downregulated in tissues of IPF patients (63). Previous studies found that S100A12 is mainly elevated in monocytes (64), which may be the cause of the above results. S100A12 may mainly exert a pro-inflammatory effect in monocytes to promote the occurrence of fibrosis. Previous reports indicate that plasma S100A12 levels are significantly negatively correlated with FEV1. Upregulation of S100A12 activates pro-inflammatory pathways and promotes fibrotic diseases (65).

S100A12, also a component of the previously reported 52-gene signature in Lancet Respiratory Medicine (66), is highly expressed in monocytes and macrophages and has been shown to modulate inflammatory pathways and promote fibrosis. This highlights the critical role of macrophages in pulmonary fibrosis, particularly in IPF and post-viral fibrosis, such as post-COVID-19 ILD (67). The interplay between macrophages and the nervous system, termed the “macrophage-nerve axis,” represents a novel area of research. This bidirectional communication involves macrophage-derived neurotrophic and neuro-inflammatory factors influencing neural activity, whereas neural signals reciprocally regulate macrophage function. For example, macrophage-driven mechanisms, such as netrin-1–mediated adrenergic processes, have been implicated in lung fibrosis (68). These findings emphasize the intricate regulatory networks involving macrophages in inflammatory and fibrotic processes. Although precision medicine approaches offer promising insights through the identification of biomarkers and endotypes, challenges related to infrastructure, financial, regulatory, and ethical barriers hinder clinical implementation (69, 70). Overcoming these obstacles is essential for advancing personalized care and improving outcomes in ILDs.

The current association of S100A12 with depression and lung fibrosis presents an intriguing possibility of linking these findings to research on the lung-brain axis (71, 72). The lung-brain axis, which explores the bidirectional communication between the lungs and the brain, may offer valuable insights into how exercise-induced changes in S100A12 expression could play a role in modulating both pulmonary and neurobehavioral functions. Further studies are needed to explore whether S100A12 is involved in this interaction and how it might contribute to the integrated response of the lung-brain axis in the context of pulmonary fibrosis and depression. In addition, the inflammation hypothesis has attracted much attention in depression (73). Lipopolysaccharide-induced inflammation gave rise to depression-like phenotype by altering BDNF-Tropomyosin receptor kinase B (TrkB) signaling in the prefrontal cortex, hippocampus, and nucleus accumbens (74). In the present study, decreased BDNF was also found in the hippocampal region of mice induced by BLM, which suggested that S100A12 may affect depression by influencing neuroinflammation and related pathways.

Furthermore, this study revealed the signaling pathways primarily enriched by genes significantly correlated with the S100A12 through KEGG enrichment analysis, providing important insights into how these genes cooperate to influence biological processes. Among these pathways, the IL-17 and NLR signaling pathway were the two most significantly enriched. The NLR protein family is a group of pattern recognition receptors that are known to mediate the initial innate immune response to cell damage and stress (75). The S100A12-Receptor for advanced glycation endproducts (RAGE) interaction has been shown to promote the release of macrophage cytokines and the generation of ROS to promote acute lung injury (76). Moreover, S100A12 has been found to induce inflammation and apoptosis in septicemia-induced ARDS by activating the NLRP3 inflammasome signaling pathway (77). NLRP3 activation increases airway resistance, reduces lung compliance, and causes distal lung epithelial remodeling in mice, thus exacerbating pulmonary fibrosis progression (78). Recent studies suggest that IL-17 subtypes are crucial for mediating acute and chronic inflammation through innate and adaptive immunity. In the context of BLM injury, the expression of IL-17A is upregulated, resulting in an increase in the expression of other pro-inflammatory cytokines such as TNF-α and chemokines such as IL-8 and CXCL5 in endothelial cells and epithelial cells, which further accelerating the formation of fibrosis (79). These results indicate that S100A12 may be involved in the occurrence and development of pulmonary fibrosis by regulating inflammatory response through IL-17 and NLR signaling pathway. This lays a solid foundation for further functional validation and mechanism studies.

The strength of this study is to explore the effects of exercise on pulmonary fibrosis and depression through animal models and behavioral experiments, suggesting that physical exercise may be an effective intervention to inhibit disease progression. In addition, we identified the key target S100A12 and its potential pathways where exercise affects pulmonary fibrosis and depression by analyzing the GEO dataset.

However, this study also has certain limitations. First, the above results are based on phenotypic studies and bioinformatics analyses. Second, the expression of S100A12 was not verified in blood and tissues of patients with IPF. Therefore, it is necessary to further verify the function and molecular mechanism of S100A12 *in vivo* and *in vitro* through genetic or pharmacological methods in the future.

Moreover, although we standardized the exercise load using treadmill running to ensure consistency in physical activity across subjects, we recognize that voluntary wheel running may more accurately reflect natural behavioral patterns of exercise. In future studies, it will be important to incorporate voluntary wheel running as a complementary exercise modality to cross-validate the current findings and further investigate the effects of different exercise behaviors on disease outcomes. This approach will help determine whether the results observed with treadmill running are consistent across various forms of physical activity, providing a more comprehensive understanding of how different exercise patterns influence the progression of pulmonary fibrosis and depression. Additionally, examining voluntary wheel running may offer insights into the role of intrinsic motivation in exercise, potentially revealing new mechanisms through which physical activity impacts disease processes.

## Conclusions

This study confirms that a high dose of BLM can induce IPF mouse model accompanied by anxiety and depression-like behaviors. The results suggest that exercise training significantly alleviates BLM-induced pulmonary fibrosis, improves lung function, and effectively mitigates anxiety-depression-like behaviors. Exercise training may exert its therapeutic effects by modulation biomarkers and signaling pathways associated with pulmonary fibrosis.

## Data Availability

The datasets presented in this study can be found in online repositories. The names of the repository/repositories and accession number(s) can be found in the article/**Supplementary Material**.

## Supplementary material

Supplementary Figure 1 7.5mg/kg BLM induces lung fibrosis and anxiety- and depression-like behaviors in mice. **(A)** Representative images of Hematoxylin-eosin staining for varying dosage of BLM. **(B)** Comparison of Ashcroft score. **(C)** Survival curve. **(D)** Motion trajectories, motion heatmap **(E)** and residence time heatmap **(F)** in the OFT of mice. Analysis of the movement velocity **(G)**, immobility time **(H)**, frequency of entrance in the central area **(I)**, and time spent in the center **(J)** during the OFT of mice. **P*<0.05, ***P*<0.01, ns *P*>0.05.

Supplementary Table 1 Primary antibody list.
