# Supplementary figures and images for: Exercise improves pulmonary fibrosis and neurological symptoms via S100A12 inhibition
Source: Front Immunol. 2025 Jun 27;16:1583827. doi: 10.3389/fimmu.2025.1583827 (PMC12245693; doi:10.3389/fimmu.2025.1583827)

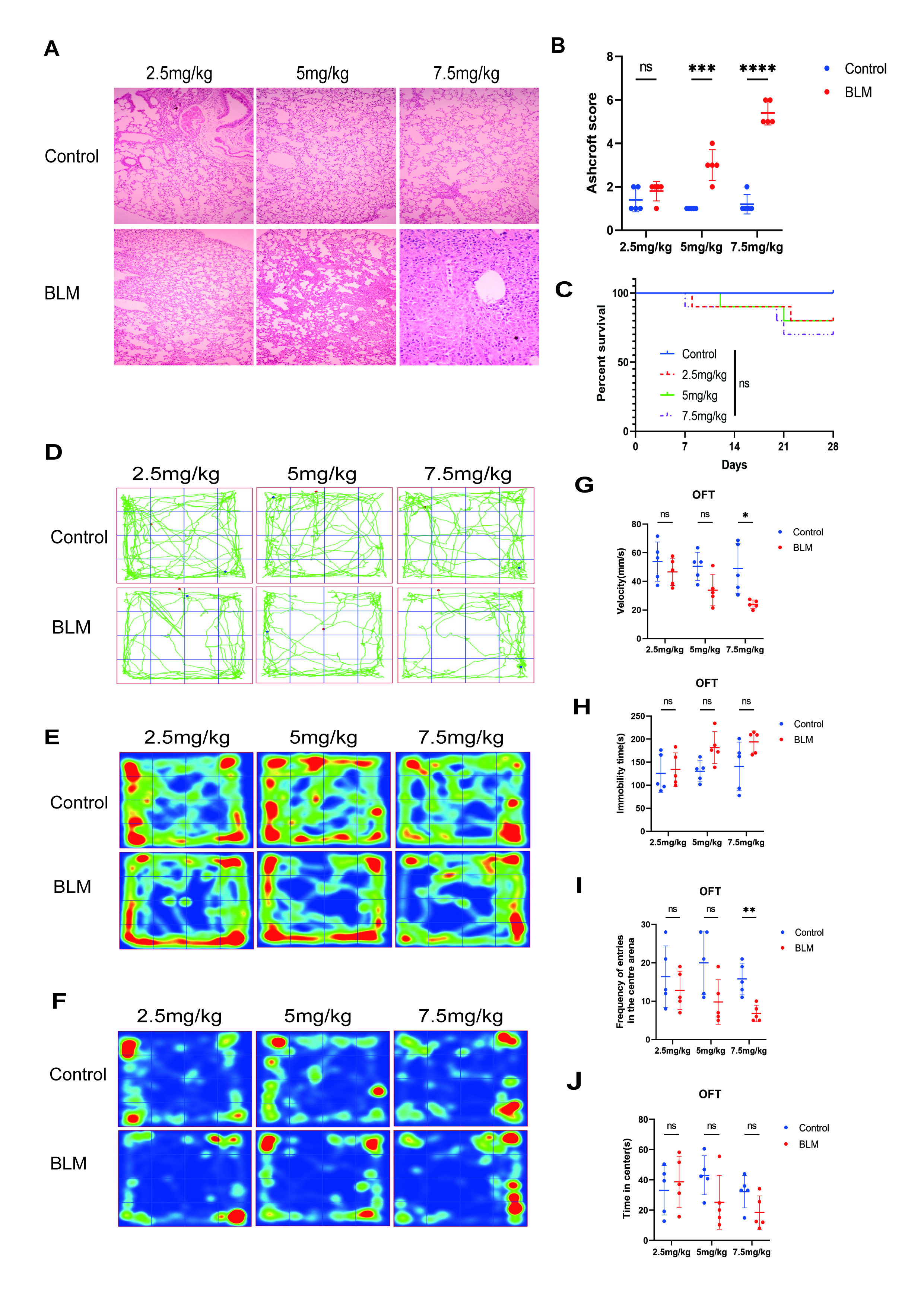

Supplement: Supplementary Figure 1 — 7.5mg/kg BLM induces lung fibrosis and anxiety- and depression-like behaviors in mice. (A) Representative images of Hematoxylin-eosin staining for varying dosage of BLM. (B) Comparison of Ashcroft score. (C) Survival curve. (D) Motion trajectories, motion heatmap (E) and residence time heatmap (F) in the OFT of mice. Analysis of the movement velocity (G), immobility time (H), frequency of entrance in the central area (I), and time spent in the center (J) during the OFT of mice. *P<0.05, **P<0.01, ns P>0.05. [file Image1.tif]
