# Supplementary material for: Exercise improves pulmonary fibrosis and neurological symptoms via S100A12 inhibition
Source: Front Immunol. 2025 Jun 27;16:1583827. doi: 10.3389/fimmu.2025.1583827 (PMC12245693; doi:10.3389/fimmu.2025.1583827)
Supplement: Supplementary Table 1 — Primary antibody list. [file Table1.docx]

Supplementary Table 1 Primary antibody list.

| Antibodies | Manufacturer | Source | Re-activity | Dilution | Catalog numbers |
| --- | --- | --- | --- | --- | --- |
| BDNF | Servicebio | Rabbit | Mouse | 1:200 | GB11559 |
| c-Fos | Servicebio | Rabbit | Mouse | 1:200 | GB12069 |
| α-SMA | CST | Rabbit | Mouse | 1:1000 | 14968 |
| Collagen Ⅰ | CST | Rabbit | Mouse | 1:1000 | 72026 |
| S100A12 | Immuoway | Rabbit | Mouse | 1:1000 | YN2246 |

BDNF, Brain-derived neurotrophic factor.
